# Catalytic Pyrolysis of Polystyrene Waste in Hydrocarbon Medium

**DOI:** 10.3390/polym15020290

**Published:** 2023-01-06

**Authors:** Konstantin I. Dement’ev, Stanislav P. Bedenko, Yulia D. Minina, Aniya A. Mukusheva, Olga A. Alekseeva, Timur A. Palankoev

**Affiliations:** 1 Topchiev Institute of Petrochemical Synthesis, Russian Academy of Sciences (RAS), 119991 Moscow, Russia; 2 Faculty of Chemical Technology and Ecology, Gubkin Russian State University of Oil and Gas, 119991 Moscow, Russia

**Keywords:** polystyrene, zeolites, catalytic pyrolysis, depolymerization, waste recycling

## Abstract

The fast catalytic pyrolysis of polystyrene in the hydrocarbon medium (light and heavy cycle oil) over zeolite catalysts at 450–550 °C was investigated. The influence of reaction conditions (medium, temperature, vapor residence time, polystyrene concentration) on polymer conversion and product distribution was studied. It was found that the polymer conversion is close to 100%, while ethylbenzene, benzene, and toluene are the main products of its transformation. The maximum yield of ethylbenzene (80%) was achieved at 550 °C, vapor residence time 1–2 s, polystyrene concentration 10%, and heavy cycle oil as the medium. The influence of zeolite topology on product distribution was explored. The possible mechanism of polystyrene pyrolysis was proposed.

## 1. Introduction

Nowadays, various polymers, such as polyethylene, polypropylene, polystyrene (PS), and others, are widely used. According to [1], approximately 390 million tons of various polymer products are manufactured per year whereby over 270 million tons of polyolefins end up as a waste on a dump [2]. The recycling of such waste is a need due to the fact that common polymers are very stable, and their bio-degradation requires much time [3]. Chemical recycling of the polymer waste can be considered the most economical and ecological way of their recovery because this approach enables obtaining high-value hydrocarbons, hence diversifying petrochemical feedstock. Various governments are taking into account these circumstances and are funding the approach [4,5]. Moreover, the EU strategical program supposes that the polymers will be displaced by the bio-degradable or totally recyclable materials in the packaging until 2030 [6]. 

PS is one of the most widely used polymers and has a high value for recycling from a practical point of view. It is applied in packaging manufacturing, various design elements, appliance frames, etc. Its recycling is very important because PS makes up a quarter (about 22%) of the total polymer waste [7]. There are some ways to dispose of polymers, such as burning, recycling, and thermocatalytic recovery. However, mentioned methods are only a partial solution [8]. Burning is the main technology for PS waste disposal nowadays [9]: whereas toxic compounds (such as benzo[α]pyrene and other polyaromatic hydrocarbons) are liberated into the atmosphere during the process, alternative technologies are extremely needed [10]. 

Various processes, such as thermal pyrolysis [11,12,13,14], plasma- and microwave-assisted pyrolysis [15,16], catalytic pyrolysis [11,12,14,17,18], hydrocracking [19], waste gasification process [4,20], and plasma-assisted gasification process [4], are developed nowadays. The thermal and the catalytic industrial units were realized in Japan, while the gasification unit «Enerkem» was built in Canada [4]. 

The catalytic pyrolysis of PS seems to be the most perspective process due to its predominant features in comparison with the above technologies: the catalytic reaction proceeds at lower temperatures (250–550 °C, while the thermal one is carried out at 300–875 °C), applies reduced residence times, requires lower amounts of thermal energy and electricity [3,12,16,18,19,20,21,22,23,24,25,26,27,28,29,30]. Moreover, not only styrene but also benzene, toluene, ethylbenzene, xylene, and other aromatic hydrocarbons required in modern industry can be obtained via the thermocatalytic process.

Various materials, such as zeolites (H-MFI [12,17,31,32], H-MTW [33], H-FAU [12,14,17,34,35], H-HEU [12], H-MOR [14,34], H-BEA [34,36]), bentonite clay [18], AL-MSU-F [37], Br-HIPS [38], Al-Al_2_O_3_ [27], CuO [39], mesoporous materials (K_2_O/Si-MWW, K_2_O-BaO/MWW, sepiolite-based MWW [22,26,38,40]) are proposed as the catalyst. Previously it was shown [41] that the thermal pyrolysis of PS in the hydrocarbon medium (such as light (LCO) and heavy (HCO) cycle oils) at 450–550 °C allows to obtain styrene with yield 84.4% and 82.5% for LCO and HCO, respectively. The role of hydrocarbon medium, as proposed solvate, is to physically separate polymer chains thus reducing side intermolecular reactions, the yield of oligomers and side products. The utilization of LCO and HCO as the reaction media also enables to convert PS waste as a feedstock for the FCC that is the largest catalytic unit in the modern petrochemistry. However, the depolymerization of the PS solutions in LCO and HCO over zeolites used as the FCC catalysts has not been studied yet. 

Taking these circumstances into account, the aim of this work is to systematically study the influence of the reaction conditions on the product distribution of the PS depolymerization in the LCO and HCO medium.

## 2. Materials and Methods

### 2.1. Materials Characterization

The commercially available polystyrene 158 K (BASF) was used as a model substance of the PS waste. See Table 1 for the main properties of the material. LCO and HCO by PJSC Nizhnekamskneftekhim were used as a hydrocarbon medium. Their characterization is shown in Table 2. Benzene (Reakhim, 99.8% purity) was also used as the medium.

The commercially available zeolite powders CBV712, CP814E*, and CBV3024E (all from Zeolyst) denoted as Y (FAU topology), β (BEA topology), and ZSM-5 (MFI topology), respectively, were used as catalysts. Their characterization is presented in Table 3. The protonic form of all zeolites was obtained by calcination in air at 550 °C for 4 h before use.

### 2.2. Polymer Dissolution

The PS dissolution in LCO and HCO was carried out in beaker at 110–120 °C under continuous stirring at 300 rpm until complete dissolution. The PS concentration was varied in the range 5–15% (hereafter, all percentages refer to weight %, unless otherwise stated). The upper concentration was limited by the laboratory setup pumpability.

### 2.3. Activity Tests

The PS depolymerization was carried out on special experimental setup with an annular gap fixed bed reactor at 450–550 °C and a vapor residence time 0.2–3.5 s. The setup detailed description was presented elsewhere [41]. Reaction conditions were chosen based on previous study [41]. The vapor residence time was calculated on the basis of the average vapor velocity of the hydrocarbon medium and the light depolymerization products under the relevant reaction conditions using Equation (1):

(1) τ=VvoidF∗Vm∗273.15T∗11−ωMm+0.5∗Conv∗ωMlp+0.5∗1−Conv∗ωMhp

where *τ* is the vapor residence time (s), *V_void_* is the void reaction volume (mL), *F* is (g × s^−1^), *V_m_* is the standard molar volume at 273.15 K (22.4 × 103 mL × s^−1^), *T* is the reaction temperature (K), *ω* is PS concentration, *M_m_* is the medium molar weight (g × mol^−1^), *Conv* is the PS conversion, *M_lp_* and *M_hp_* are the average molar weights of the light and heavy PS recycling product, respectively. Insofar as PS concentration (*ω* < 0.15) was low, the vapor residence time was determined mostly by the molecular weight of the medium (term (1 − *ω*) × *M_m_*^−1^), while the contribution of the light products of depolymerization (term 0.5 × *Conv* × *ω* × *M_lp_*^−1^) was significantly less. The contribution of oligomers to the vapor residence time calculation (the term 0.5 × (1 − *Conv*) × *ω* × *M_hp_*^−1^) was less than 1% caused their high molecular weights and low yields compared to that of the light depolymerization products, and therefore, this term was not taken into account. The vapor residence time was changed by varying the feed rate and void volume of the reactor.

The gaseous reaction products were collected and then analyzed by GC (Meta-Chrom Crystallux-4000M with TCD) equipped with a packed column CaX (3 m, 3 mm) to determine H_2_, O_2_, N_2_, and CH_4_ concentration while a capillary column Agilent HP-PLOT/Q (30 m, 0.32 mm, 20 μm) was used for other hydrocarbons. Ar and He were chosen as a carrier gas for the CaX and a HP-PLOT/Q, respectively. The liquid product distribution was also estimated by GC (Meta-Chrom Crystallux-4000M with FID) equipped with an Agilent DB-2887 capillary column (10 m, 0.53 mm, 3 μm). The control samples of the liquid hydrocarbon product were additionally analyzed by GC-MS (Thermo Focus DSQ II GC-MS) equipped with a Varian VF-5 ms capillary column (30 m, 0.25 mm, 0.25 μm) to identify hydrocarbon composition. The mass-spectrum database NIST/EPA/NIH 15 was used for component identification.

The material balance was calculated for each experiment using the data obtained; the total weight of the products obtained in each experiment was more than 99% that of the initial feed. The yield of the PS depolymerization products was calculated on the polymer basis using Equation (2):

(2) $$Y_{i}=\frac{{\left(Y_{i}\right)}_{mix}-{{(Y}_{i})}_{blank}*(1-\omega )}{\omega }$$

where *Y_i_* is the yield of the *i*-product, (*Y_i_*)*_mix_* is the yield of the *i*-product in the PS conversion in the hydrocarbon medium, and (*Y_i_*)*_blank_* is the yield of the *i*-product in the hydrocarbon medium conversion.

The PS conversion was estimated using Equation (3):

(3) $$Conv=\frac{{(Y}_{benz}+Y_{tol}+Y_{ebenz}+Y_{styr}+Y_{mstyr})}{\omega }*100\%$$

where *Y_benz_*, *Y_tol_*, *Y_ebenz_*, *Y_styr_*, and *Y_mstyr_* are the yield of benzene, toluene, ethylbenzene, styrene, and *α*-methylstyrene, respectively; *ω* is PS concentration.

Although the heavier products of the PS depolymerization were also detected by GC-MS, however, their yield was difficult to estimate because their chromatographic peaks interfered with those of the hydrocarbon medium components. The selectivity toward *i*-product was computed using Equation (4):

(4) $$S_{i}=\frac{Y_{i}}{Conv}*100\%$$

where *Y_i_* is the yield of the *i*-product, and *S_i_* is the selectivity of the *i*-product.

The data were carefully examined, and statistical analysis was carried out to confirm its reproducibility. The coke yield for the zeolite *Y* is about 4–6% at the whole test, and these values are close to it for the blank test. For other zeolites, the coke yield was not measured.

## 3. Results and Discussion

### 3.1. Conversion of Pure LCO and HCO over Y Zeolite

**Figure 1:**
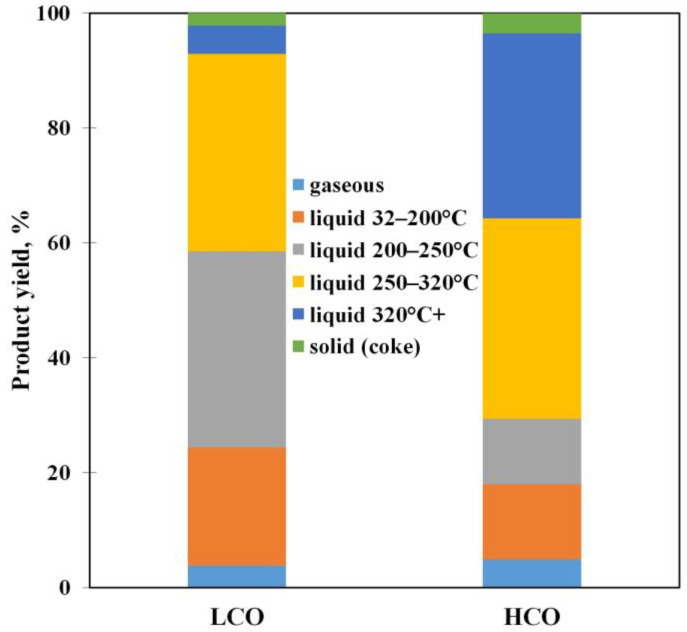
The product distribution of the LCO and HCO conversion at 550 °C and WHSV 85 h^−1^.

A series of hydrocarbon medium (pure LCO and HCO) conversion tests were carried out over Y zeolite in the range of studied reaction conditions. The conversion of both medium types was estimated analogically to [41] by the gaseous and liquid (with a boiling point below 200 °C) product yields. The conversions at 550 °C and WHSV 85 h^−1^ are 15.1% and 14.2% for LCO and HCO, respectively. The product distributions obtained are shown in Figure 1. The gaseous product mainly consists of C_3_–C_4_ hydrocarbons, while the liquid one contains benzene, toluene, ethylbenzene, and xylenes (*o*-, *m*-, and *p*-isomers) (Table 4).

### 3.2. The Depolymerization of PS in LCO and HCO Medium over Y Zeolite

**Figure 2:**
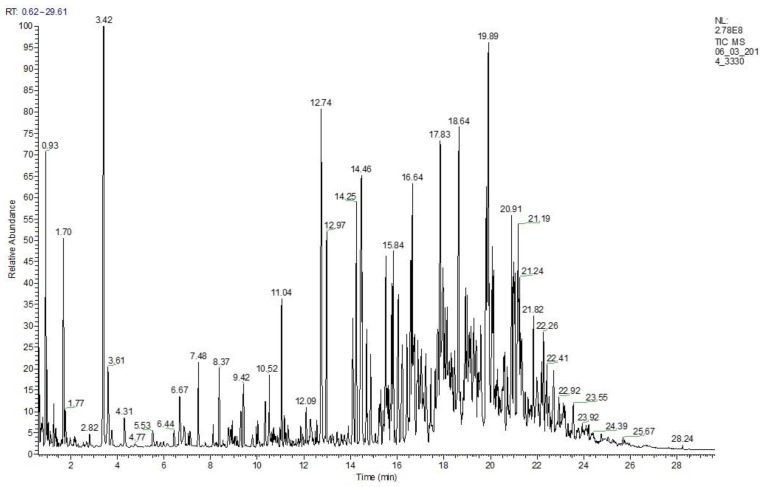
The GC–MS spectrum of the liquid product of the PS depolymerization in LCO medium over the Y zeolite at 550 °C, WHSV 85 h^−1^, and PS concentration 10%.

Ethylbenzene is the main product of the PS depolymerization in the range of conditions studied for both mediums, while benzene, toluene, and xylene can be considered major side products of the reaction. The yield of the gaseous product varies from 4.7% to 8.5% based on the PS solution weight. The secondary cracking reactions of both the PS conversion products and the hydrocarbon medium components obviously lead to gas production, which is confirmed by the pure medium conversion test. The gas of PS depolymerization in both media consists of light olefins (ethylene and propylene), *i*-butane, and methane formation, which can be explained by dealkylation and cracking as well. Benzene, toluene and ethylbenzene are identified by GC-MS (Figure 2 and Table 5). There are also other aromatics, such as naphthalenes, phenanthrenes, anthracenes, and their derivates, which are components of LCO. The liquid product composition of the PS depolymerization is shown in Table 6.

It should be emphasized that the xylene yield during the pure medium conversion is higher than their yield during the PS depolymerization in the hydrocarbon media. This observation clearly confirms that xylene formation is reduced or they are spent in the secondary reactions. We assume that xylenes, which are obtained via the cracking of media, can react with benzene, which is mainly produced via PS decomposition. The benzene-xylene transalkylation yielding toluene seems to be the most likely secondary transformation of xylenes. Due to ethylbenzene being the main reaction product, the process may be considered the two-stage one. The thermal cracking of PS is the first stage, where the styrene and other unsaturated products are obtained via radical depolymerization. In the second stage, the unsaturated products are adsorbed by the acid sites of the catalyst and then saturated during the hydrogen transfer reactions. As a result, the primary reaction product, such as styrene in the PS depolymerization case, undergoes saturation of the double bond, and the saturated ethylbenzene is generated. Ethylbenzene, in turn, can be spent on benzene and ethylene generation via the dealkylation reaction and on xylene synthesis via the isomerization route. Meanwhile, other products of the PS depolymerization (such as benzene and toluene) are also reactive over the Y zeolite; then, they can interact with ethylbenzene or each other.

### 3.3. Effect of Temperature

**Figure 3:**
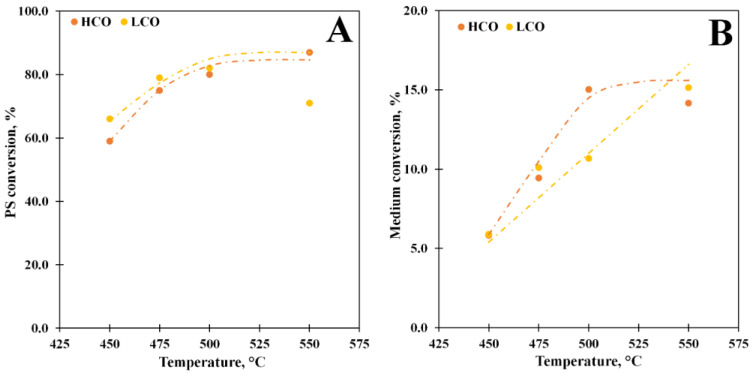
The conversion vs. temperature at WHSV 85 h^−1^ for the PS solutions in LCO (15% PS) and HCO (10% PS) (**A**) and for pure LCO and HCO (**B**).

A series of tests with different temperatures in the range of 450–550 °C were carried out to estimate temperature influence on the PS conversion and product distribution in the LCO and HCO media. The data obtained are shown in Figure 3A. Temperature growth from 450 °C to 550 °C leads to a significant increase in the PS conversion (from 59% to 87%) in the HCO medium. For the PS solution in LCO, the maximum yield of the PS conversion is obtained at 500 °C. The differences in the data obtained can be explained via PS solution behavior. Previously it was shown that the PS-medium affinity defined by the Hildebrand solubility parameter greatly affects the PS depolymerization and the distribution of the PS depolymerization products [41]. This parameter is equal to the square root of the cohesive energy density, and it is used to estimate the degree of interaction between two materials. According to the data, the PS-LCO interactions are stronger than PS-HCO ones, which led to higher PS conversion in LCO in thermal pyrolysis. It is obvious that the strong interactions between LCO and PS account for the differences observed in the catalytic PS conversion: we propose that the molecules of the medium and the primary reaction product compete with each other for the acid site of the catalyst surface. The data obtained for the conversion of pure LCO and HCO (Figure 3B) demonstrate that the LCO conversion grows linear with temperature, while the HCO one increases only until 500 °C and does not change further, which confirms our assumption about the competition between PS and medium molecules during the catalytic conversion.

**Figure 4:**
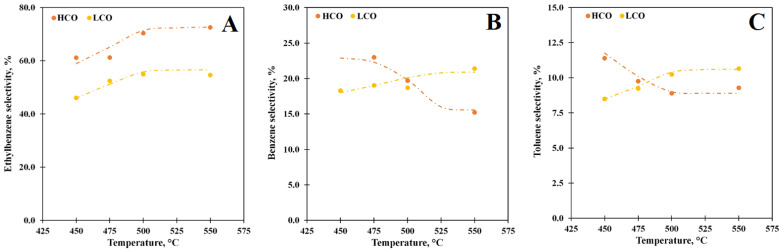
The ethylbenzene (**A**), benzene (**B**), and toluene (**C**) selectivity vs. temperature at WHSV 85 h^−1^ for the PS solutions in LCO (15% PS) and HCO (10% PS).

Ethylbenzene is the main product of the PS depolymerization in the temperature range studied, and its selectivity grows with temperature for both media (Figure 4A): the highest selectivity for ethylbenzene equals 72.5% and 54.6% at 550 °C for the HCO and LCO solutions, respectively. In the case of the LCO solution, the styrene dimers and biphenyl butanes are also determined in the product composition, which explains the reduction of the ethylbenzene yield for this solution in comparison with the HCO ones. It should be noted that the selectivity curves for both media are equidistant; hence, we can presume that there is a uniform mechanism for ethylbenzene synthesis via the PS depolymerization process catalyzed by the Y zeolite. Benzene was the second reaction product of the PS depolymerization catalyzed by the Y zeolite for both media. The selectivity curves vs. temperature for them are shown in Figure 4B. When PS is converted in LCO, the benzene selectivity curve has the same variation versus temperature as one of ethylbenzene: the maximum selectivity toward benzene (21.4%) is obtained at 550 °C. At the same time, the benzene selectivity curve has a downing trend for the depolymerization of the PS solution in HCO: it has a plateau at 450–475 °C with a value of 23.0% and then decreases with the temperature growth. It is the secondary transformations of ethylbenzene that are responsible for the high yield of benzene, while its yield was within 0.2% when the pure media converted over the Y zeolite. The toluene selectivity is about 10% for both media (Figure 4C). The temperature increase leads to the growth of selectivity up to 10.7% at 550 °C in LCO, which may be associated with the xylene-benzene transalkylation reaction that is confirmed by the decrease in the xylene yield for the PS solutions in comparison with the pure media conversion. For the case of the PS-HCO depolymerization, the toluene selectivity drops down from 11.4% at 450 °C to 9.3% at 550 °C due to the secondary transformation of toluene. Xylenes and styrene were the minor products for both media, while *α*-methylstyrene, C_13,_ and C_14_ hydrocarbons were also obtained as the products of PS-LCO depolymerization.

### 3.4. Effect of the Vapor Residence Time

**Figure 5:**
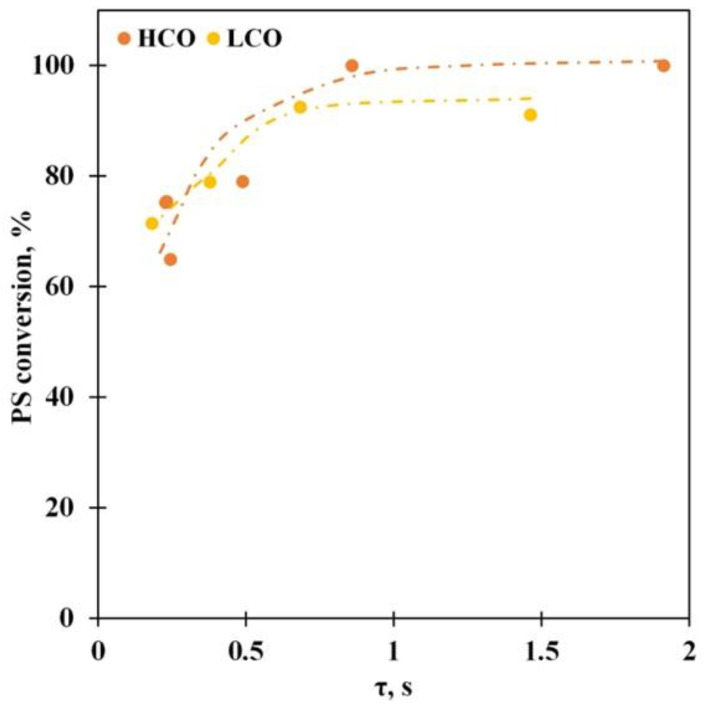
The PS conversion vs. the vapor residence time for the PS solutions in LCO (15% PS) and HCO (10% PS) at 550 °C.

The experiments with the different vapor residence times make it possible to estimate process behavior more thoroughly. An increase in the residence time leads to growth of the conversion (Figure 5): the PS conversion reaches 93% at the residence time, equal to or more than 0.5 s for the PS solution in LCO; however, it does not grow further and stays constant with further increase in the residence time. At the same time, conversion rises to 100% at the residence time higher than 1 s for the PS solution in HCO. We assume that conversion remaining at the same level may be due to the competition between molecules of media and PS depolymerization products. Acceleration of secondary condensation reactions, which can yield heavy products, is also possible.

**Figure 6:**
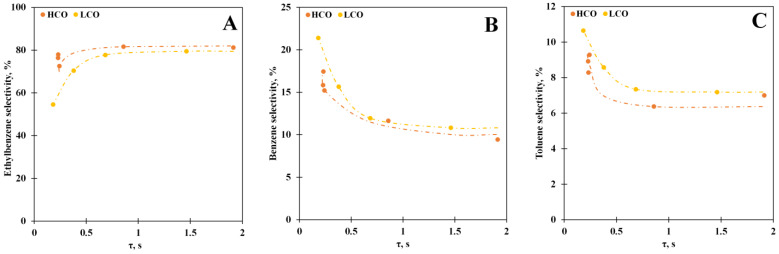
The ethylbenzene (**A**), benzene (**B**), and toluene (**C**) selectivity vs. the vapor residence time for the PS solutions in LCO (15% PS) and HCO (10% PS) at 550 °C.

In addition, an increase in the vapor residence time favors the ethylbenzene selectivity growth (Figure 6A), whereas the amounts of toluene (Figure 6B) and benzene (Figure 6C) in the reaction products are reduced. The maximum ethylbenzene selectivity equals 80% for both media. It is obvious that the vapor residence time influences the hydrogen transfer reactions, and its increase stimulates the transfer; however, this influence reaches a limit at values above 1 s; the decrease in the benzene and toluene selectivity is caused by the secondary reactions such as dealkylation and cracking that is confirmed by growth of the gaseous product yield. It must be noted that the selectivity toward the secondary products is reduced for the HCO medium reaction in comparison with the PS-LCO depolymerization case. We propose that this observation also emphasizes the competition between PS and LCO medium molecules for the adsorption on the catalyst active sites.

### 3.5. Effect of the PS Concentration

**Figure 7:**
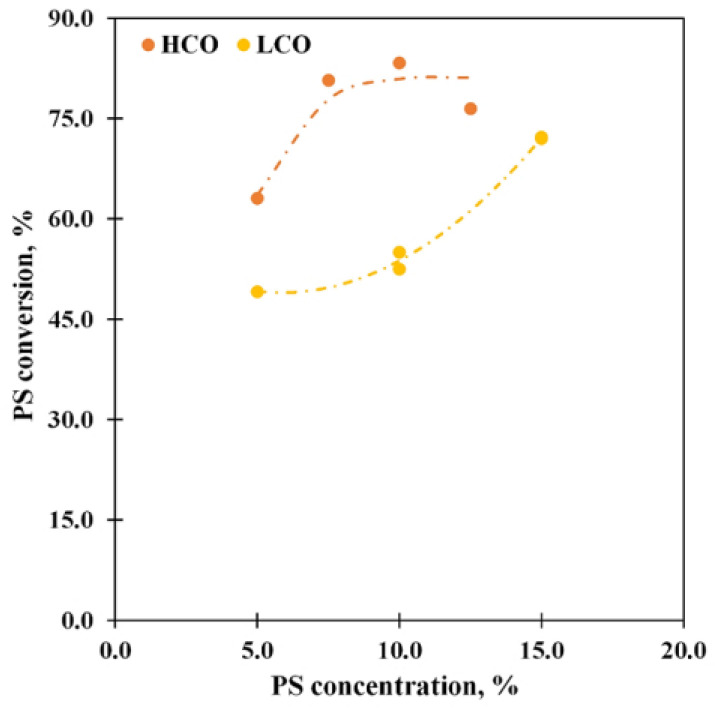
The PS conversion vs. the PS concentration at 550 °C and WHSV 85 h^−1^.

**Figure 8:**
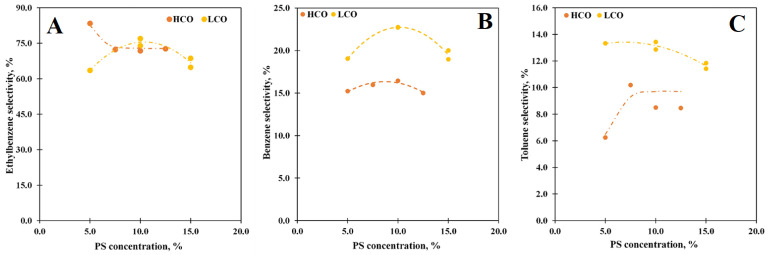
The ethylbenzene (**A**), benzene (**B**), and toluene (**C**) selectivity vs. the PS concentration at 550 °C and WHSV 85 h^−1^.

A series of experiments with different PS concentrations were carried out due to the PS concentration greatly affects the conversion and product distribution via the thermal depolymerization of the PS solutions in LCO and HCO [41]. The increase in PS concentration generally influences the PS conversion (Figure 7) and the product distribution (Figure 8), likewise to the thermal depolymerization.

The curves obtained for the PS conversion are similar to the curves for the thermal pyrolysis [41]: the conversion rises with the PS concentration increase and grows to 72.2% at 15% concentration for the LCO solution; the maximum of PS conversion (83.3%) is observed at 10% for the PS-HCO depolymerization case. Unfortunately, the PS solutions in LCO with a concentration over 15% are too viscous, and setup pumpability limits their study, while for the PS solution in HCO increase in the concentration leads to the fall of the conversion. It is obvious that the interactions between PS and the molecules of the media greatly affect the catalytical depolymerization process as in the thermal pyrolysis case: since LCO has a higher affinity toward PS than HCO, an increase in the PS concentration is more significant for the depolymerization of the PS solutions in LCO and the PS-LCO interactions are mostly physical. Moreover, lower values of the conversion for the PS-LCO case are also attributed to the competition between PS and LCO medium molecules for the adsorption on the catalyst active sites that is in accord with the data described above.

The changing of the PS concentration does not modify the product distribution dramatically: ethylbenzene stays the main product (Figure 8A), while benzene (Figure 8B) and toluene (Figure 8C) are still the second and third products, respectively. For the PS solution in LCO, the ethylbenzene selectivity curve has a maximum of 77% at 10% concentration. At the same time, for the PS-HCO case, the curve decreases from 83.4% at 5% to 72.3% at 7.5% and does not significantly change with a further concentration increase. Apparently, the rising of the PS concentration facilitates the side reactions of ethylbenzene that lead to its selectivity failing. In addition, the hydrogen transfer reactions are also inhibited, and the styrene selectivity grows as a result. The benzene selectivity curves have a maximum of 22.7% and 16.4% at a concentration of 10% for the PS-LCO and PS-HCO solutions, respectively. The selectivity toward toluene in LCO also has a maximum of 13.4% at a concentration of 10%, while for HCO, the curve increases to 10.2% at 7.5% concentration and then slightly reduces with further growth of the PS concentration in the solution.

**Figure 9:**
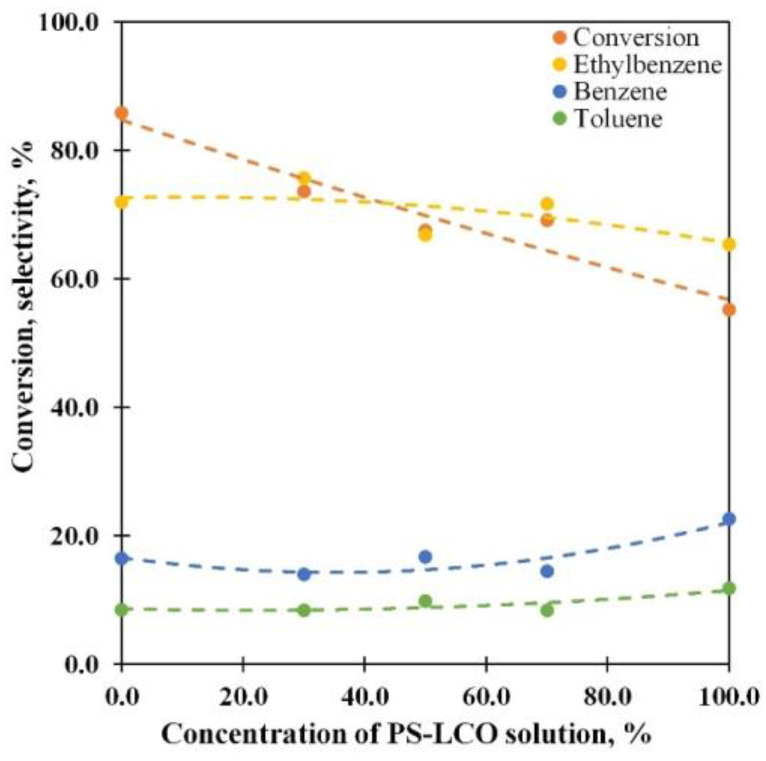
The PS conversion and main product selectivity vs. the PS-LCO solution concentration at 550 °C and WHSV 85 h^−1^ for mixed 10% solutions of PS in LCO and HCO.

As supplementary to the former, a series of experiments with the mixed medium were carried out (Figure 9). For this, the solutions with a 10% concentration of PS were mixed together with different ratios. An increase in the PS-LCO solution in the feedstock results in the conversion and ethylbenzene selectivity decreasing. At the same time, the selectivity toward benzene and toluene moderately grows. Therefore, it is possible to control the PS conversion and product distribution via the changing of medium composition.

### 3.6. Proposed Mechanism of Catalyzed Depolymerization

The PS depolymerization over the catalyst may proceed by two different mechanisms. The first consists of two stages: primarily, the polymer chains are thermally shredded then the products of the PS depolymerization (styrene, its dimers, and trimers) are subsequently converted over the acid sites of the zeolite. The second mechanism supposes adsorption of the PS chains on the sites with the formation of the carbocations, which further undergo β-elimination. Additional tests were carried out to discriminate the more probable mechanism of the catalyzed depolymerization.

First of all, it was shown in [41] that the thermal pyrolysis proceeds very fast: the PS conversion reaches 100% at the vapor residence time, even less than 0.5 s. The styrene yield equals 50% at the residence time 0.05–0.06 s in the experiment with benzene as the reaction medium. Therefore, the substances that contact the catalyst are the products of the PS depolymerization, not its chains.

The reaction of ethylbenzene and styrene was studied at the depolymerization conditions to estimate the product distribution of the secondary reactions (Table 7). It must be noted that both hydrocarbons are stable without a catalyst in the reactor. At the same time, ethylbenzene is dealkylated, yielding benzene and isomerized into xylenes, which in turn react with benzene and produce toluene over a zeolite catalyst. Nevertheless, the ethylbenzene conversion is less than 15% at the low residence time. On the other hand, the styrene conversion over the catalyst is close to 100%: ethylbenzene is the main product of the reaction, and the hydrogen transfer reactions typical for the zeolite catalysis seem to be the way for its generation. Styrene is known to have an unsaturated bond in the aliphatic chain; when this bond is protonated by the acid sites of a zeolite, resonance-stabilized carbocation is produced as a result. The formation of such carbocation is much more favorable than the ring-protonation, which explains why styrene is more reactive than ethylbenzene. In general, the styrene transformation proceeds in the same way as ethylbenzene. However, high selectivity toward toluene is also caused by an additional reaction between styrene and ethylbenzylic carbocation, yielding dimeric carbocation, and toluene and C_9_ hydrocarbons are produced as a result of its proceeding decomposition. This way, toluene generation via the styrene conversion has been confirmed by the experimental data on its transformation in the benzene medium where C_9_ hydrocarbons are obtained as the product. It should be emphasized that the product distribution of styrene transformation and polystyrene depolymerization are alike. The dimers and trimers generation via the initial PS cracking may cause the high yield of benzene and toluene in the process due to their secondary transformation leading to benzene, toluene, and C_9_ hydrocarbon formation.

**Figure 10:**
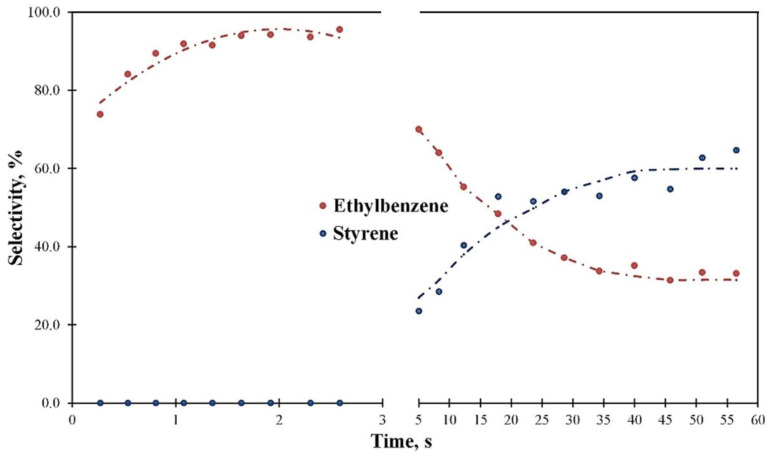
The main product selectivity vs. time on stream at 550 °C and WHSV 85 h^−1^ for the 10% PS solution in HCO.

Thus, the hydrogen transfer reactions have a great contribution to the product distribution at the PS decomposition in the media over the Y zeolite. The experiment with impulse feed injection for 1 min without the catalyst regeneration was carried out to estimate the influence of the hydrogen transfer reaction on the selectivity (Figure 10). According to the data obtained, the time on stream greatly affects the reaction selectivity: during the first 9 impulses, the selectivity toward ethylbenzene increases to 95% while the styrene selectivity is close to 0%; after 5 s on stream, the selectivity for ethylbenzene decreases. At the same time, the styrene selectivity starts to grow at 5 s from approximately 20% to 67.5% at 1 min of the stream. Styrene becomes the main reaction product after 15 s on stream. It is obvious that the catalyst undergoes deactivation by the coke deposits and the reaction does not progress completely and primarily stops at the first stage—the thermal decomposition. Nevertheless, full deactivation of the catalyst is not obtained at the studied conditions. Probably, the strongest sites are deactivated at the initial moment of the reaction, and then other strong sites steadily lose their activity with the increase in the time on stream. At the same time, some quantity of sites with low or medium strength is still available for the adsorption; thus, the catalyst keeps a partial activity during the test. It should be noted that the Y zeolite already loses activity at cat-to-oil ratios of less than 1 during the hydrocarbon cracking reaction, whereas it is highly stable during the PS decomposition in the hydrocarbon media since styrene is detected only after the high quantity of feed (about 36-fold than the catalyst mass) is passed over the catalyst.

**Figure 11:**
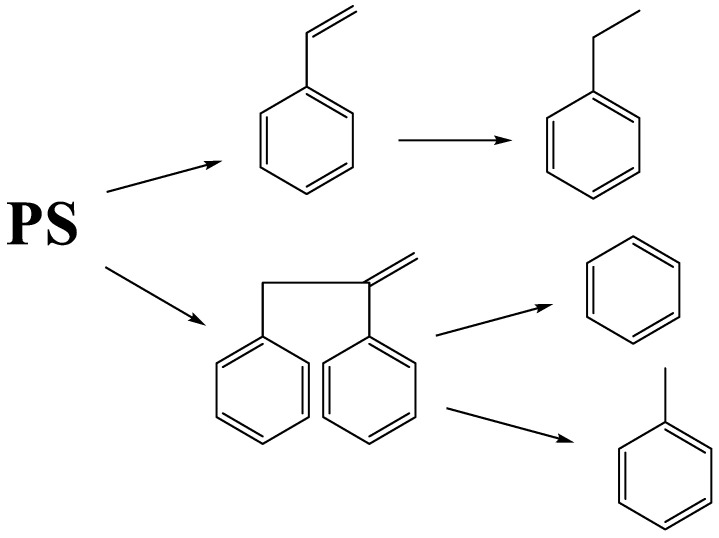
Scheme of the PS decomposition ways.

According to obtained data and previous work [41], we proposed the simplified reaction scheme that is shown in Figure 11. This scheme demonstrates the ways of the main product generation via the PS depolymerization and does not take into account the transformation of the media.

### 3.7. The Comparing of Zeolite Topology Influence

The process of the PS depolymerization was studied over various (Y, β, and ZSM-5) catalysts in the HCO medium to estimate catalyst influence on the product distribution. The comparison was carried out at the complete substrate conversion (Table 8).

The product composition is the same for all types of catalysts; however, the change of zeolite topology enables variation of their distribution: ethylbenzene is the main product for the Y zeolite, while for the ZSM-5 and β, it is benzene. It is obvious that the topology of the catalyst is a key to reaction selectivity control. As known, these zeolites have different pore structures: the Y has the widest pores (only molecules with 7.35 Å diameter can diffuse there), the ZSM-5 has the narrowest (4.5 Å), and for the β, an effective pore size equals 5.95 Å [42]. The shape selectivity and molecular sieve effect are the main features of zeolites [43], and it is the features that can explain the data obtained. Bulky products such as ethylbenzene are obtained when the reaction is carried out over the Y zeolite with the widest pores. At the same time, the selectivity toward α-methylstyrene and styrene dimers is the lowest for this catalyst due to these molecules can reach the active sites during the diffusion into inner pores. For the ZSM-5, the opposite product distribution is observed: the shape selectivity and molecular sieve effect limit the PS transformation and inner diffusion of bulky products that lead to the highest yield of benzene and toluene, which can easily diffuse from the ZSM-5 pore and C_9+_ hydrocarbons, which cannot be obtained on the active sites due to diffusion limitations. The β has a broad product distribution, and the selectivity toward benzene, toluene, ethylbenzene, and xylenes are almost the same because of medium pore size. The data obtained are in accordance with the data published for the shape selectivity and molecular sieve effect of the zeolites and with the works where this phenomenon is studied for some other reactions [44,45,46,47].

## 4. Conclusions

The PS depolymerization in the hydrocarbon media (light and heavy cycled oil) catalyzed by zeolites has been studied. The effect of the reaction conditions (temperature, vapor residence time, PS concentration) has been explored. It was shown that the temperature increase from 450 to 550 °C leads to significant growth of the PS conversion (from 59% to 87%) in the HCO medium. For the PS solution in LCO, the maximum conversion equals 82% at 500–550 °C. It was shown that the vapor residence time affects the product distribution: the selectivity toward ethylbenzene rises to 80% at the vapor residence time 1 s or higher while selectivity toward benzene and toluene declines from 20–22% to 10% and from 10–12% to 6%, respectively. At the optimal conditions (550 °C, the residence time 1–2 s, the PS concentration 10%, and the HCO media), the yield of ethylbenzene is about 80%, benzene—12.7%, toluene—6.3%, while the conversion nears 100%.

As shown, the PS decomposition is a two-stage process: the first one is the thermal cracking of the PS chains yielding the primary products (such as styrene, its dimers, and trimers) that are further converted over the acid sites of zeolites on the second stage.

Zeolite topology’s influence on product distribution has been explored. Ethylbenzene is the main product of the PS decomposition over the wide-pore Y zeolite, benzene is the main one over ZSM-5, while β has the broad product distribution. The topology of the zeolite is a key to the selectivity control due to shape selectivity and molecular sieve effect.

The obtained data may be used to develop the PS decomposition process for the synthesis of high-octane gasolines or aromatics in the future.

## Figures and Tables

**Table 1:** PS properties.

	Value
Density, kg/m^3^	1048
Melting point, °C	180–260
Molecular weight, kg/mol	278
Dispersity	2.3

**Table 2:** Hydrocarbon medium characterization.

	LCO	HCO
Density, kg/m^3^	905	1006
Molecular weight, g/mol	178	249
Fractional composition, % wt.		
Initial boiling point, °C	133.9	154.2
10, °C	217.1	297.9
30, °C	238.8	337.7
50, °C	256.6	366.8
70, °C	280.4	398.6
90, °C	305.5	448.8
95, °C	319.4	472.1
Final boiling point, °C	345.7	514.9
Group composition, % wt.		
Saturates	20.6	14.1
Monoaromatics	16.5	2.2
Diaromatics	47.5	10.3
Polycyclic aromatics	11.0	65.0
Resins	4.4	5.1
Asphaltenes	0.0	3.3
Hildebrand solubility parameter [41], MPa^1/2^	19.1	20.1

**Table 3:** Zeolite properties.

	Commercial Name	Si/Al	S, cm^2^/g
**Y**	CP814E*	12.5	680
**β**	CBV712	6	730
**ZSM-5**	CBV3024E	15	405

**Table 4:** The component distribution of gaseous and liquid products obtained via the conversion of pure LCO and HCO at 550 °C and WHSV 85 h^−1^.

Component	LCO, %	HCO, %
H_2_	-	0.1
C_1_	0.4	0.5
ΣC_2_	0.5	0.6
ΣC_3_	1.0	1.4
ΣC_4_	1.9	2.3
Benzene	0.1	0.3
Toluene	1.4	1.1
Ethylbenzene	1.2	0.3
Σxylenes	2.9	1.7

**Table 5:** The GC-MS spectrum component composition of the liquid product of the PS depolymerization in LCO medium over the Y zeolite at 550 °C, WHSV 85 h^−1^, and PS concentration 10%.

Component	Retention Time, min
Benzene	0.93
Toluene	1.70
Ethylbenzene	3.40
Xylenes	3.60–4.00
Styrene	4.31
*α*-methylstyrene	7.48
Naphthalene	11.04
Methylnaphtalenes	12.50–13.00
Ethylnaphtalenes	14.00–15.00
C_3_-naphtalenes	15.50–16.50
Methylbiphenyls	16.55–17.00
Ethylbiphenyls	17.50–18.00
Phenanthrene	18.64
Styrene dimer	18.81
Methylphenanthrenes and methylanthracenes	19.80–20.10
Ethylphenanthrenes and ethylanthracenes	20.90–21.30
C_3_-phenanthrenes and C_3_-anthracenes	22.20–22.50
Styrene trimer	24.40

**Table 6:** The liquid product distribution for the PS conversion in LCO and HCO at 550 °C and WHSV 85 h^−1^.

Component	LCO, %	HCO, %
Benzene	21.4	15.2
Toluene	10.6	9.3
Ethylbenzene	54.6	72.5
Styrene	traces	-
Styrene dimers	traces	-

**Table 7:** The product distribution of ethylbenzene and styrene reaction at 550 °C and WHSV 85 h^−1^.

Hydrocarbon	Ethylbenzene		Styrene	
Catalyst	-	Y	-	Y
Benzene	0.1	10.8	0.1	11.4
Toluene	0.2	1.9	0.1	6.4
Ethylbenzene	93.2	84.8	0.1	72.1
Styrene	-	-	95.7	6.7

**Table 8:** The product of the PS depolymerization in HCO over various zeolites.

Product	Zeolite		
	Y	β	ZSM-5
Benzene	11.2	31.2	53.3
Toluene	9.8	27.2	23.4
Ethylbenzene	77.8	19.7	2.7
ΣXylenes	1.1	20.9	9.8
ΣC_9+_ hydrocarbons	0.1	1.0	10.8

## Data Availability

The datasets generated for this study are available on request to the corresponding author.
